# Dilution of rice with other gluten free grains to lower inorganic arsenic in foods for young children in response to European Union regulations provides impetus to setting stricter standards

**DOI:** 10.1371/journal.pone.0194700

**Published:** 2018-03-16

**Authors:** Manus Carey, Emily Donaldson, Antonio J. Signes-Pastor, Andrew A. Meharg

**Affiliations:** 1 Institute for Global Food Security, Queen’s University Belfast, Belfast, Northern Ireland; 2 Department of Epidemiology, Geisel School of Medicine, Dartmouth College, Lebanon, New Hampshire, United States of America; Stony Brook University, Graduate Program in Public Health, UNITED STATES

## Abstract

There has been an increasing realisation that young infants are exposed to elevated concentrations of the carcinogen inorganic arsenic, relative to adults. This is because many infant food products are rice based, and rice is ~10-fold elevated in inorganic arsenic compared to most other foods. The European Commission (EC) has acted on this concern setting stricter standards for infants, 100 μg of inorganic arsenic per kg of food (100 μg/kg), as compared to adults (200 μg/kg), for rice based foods, a law that was brought into place in 1^st^ January 2016. Here we investigate how this law has impacted on inorganic arsenic in baby food products in the UK market, and compare the findings to previous baby food surveys taken before and just after the law came into place. We find that for a wide range of UK infant products that the new regulations are being adhered to, with all samples surveyed, being under 100 μg/kg inorganic arsenic. The prevalence of pure rice products had decreased in the UK, and there appears to be careful sourcing of the rice used in these products to ensure conformity with regulations. There has been an increased presence of mixed cereal products, with rice and maize as the main ingredient, appearing on the UK market, with varying rice contents for infant porridges, cakes and mueslis, with the latter being a relatively innovative product for infant foods. There was a highly significant correlation (P<0.0001) between rice content and inorganic arsenic concentration across all infant foods. When UK infant rice cakes, breakfast cereals and porridges were compare to their general, i.e. not labelled specifically for being for infant consumption, equivalent it was found that the adult foods generally exceeded the 100 μg/kg inorganic arsenic standard for infant foods. Thus, infants should not be given rice products not specifically labelled as being for them if a lower inorganic arsenic diet is to be maintained.

## Introduction

Rice is widely used as an ingredient in weaning products [1]. However, inorganic arsenic is a non-threshold carcinogen that is found circa. 10-times higher in rice than in other staple foods [2]. Inorganic arsenic, as well as being a carcinogen, also impacts on child I.Q., growth rates, and immune development, amongst other effects, at concentrations that can be found in the diet [3–6]. Children are particularly more exposed to food-borne toxins, such as inorganic arsenic, as they consume circa. 3-times as much food on a body mass basis as adults [7]. Signes-Pastor et al. [7] found that when infants’ urine was sampled before and after weaning that inorganic arsenic and its metabolites monomethylarsonic acid (MMAA) and dimethylarsinic acid (DMAA), with DMAA being also the other major arsenic species in rice [8], increased 4.5-fold after weaning. Surveys of arsenic in rice based weaning/baby/infant foods have led to growing concern regarding the exposure of infants to arsenic [7, 9, 10].

An assessment of the risks posed by rice as an exposure route of inorganic arsenic to infants [11] has led to the European Union introducing new laws on the 1^st^ January 2016, setting the standard for inorganic arsenic in rice based products intended for young children, those below 5-years of age, at 100 μg of inorganic arsenic per kg of food (100 μg/kg) [12]. This is half the standard for polished (white) rice, and a third of that for puffed rice [12]. When arsenic in food containing rice marketed at young children, less than 5-years old, was surveyed in the UK just after the new standards were set, ~three-quarter of products were above the legal threshold [7]. Levels had in fact increased from a pre-ban survey [7, 13]. Typical baby foods that may be elevated in inorganic arsenic are rice porridge, rice crackers/wafers, and puffed rice cereals [7, 13]. However, these also have general, i.e. not labelled specifically for babies, counterparts, and the stricter EU regulations only apply to products labelled specifically for consumption by young children [12]. This constitutes a loophole, and unsuspecting parents have a choice between to almost identical products, but one labelled for children and one labelled for general consumption, and with a potential divergence in inorganic arsenic content. Also, studies have focussed on the UK [7, 13], with few surveys of inorganic arsenic in baby foods elsewhere in Europe, and all of which were before the new regulations of 2016 [14–16].

In this present study, we set out to establish inorganic arsenic levels in UK baby foods, as well as their general, i.e. not specifically labelled as being for infant consumption, equivalents for the UK. This was to observe how the new EU laws were being enforced, and to identify if specific children’s products had diverged from generic porridges, grain cakes and breakfast cereals. The findings of the survey were also compared concentrations found in previous investigations [7, 13]. The results were interpreted with respect as to what is the best dietary advice to give parents of young children, and how to avoid products with higher exposures, and strategies for further reducing children’s exposure to inorganic arsenic.

## Materials and methods

### Sampling strategy

For the current survey, samples of individual products were sampled from separate retail outlets, to give duplicate or triplicate samples. Date of sample purchase, location and formulation details are (S.I. S1 File). The sampling strategy was to go to major (ASDA, Boots, Dunne’s, Holland & Barrett, Marks & Spencer, Sainsbury’s) and minor retailers to purchase 3 (in a minority of cases only 2 were available, see S.I. S1 File “product averages” tab for N) replicates of each individual rice based product available in baby food sections. In total, 24 stores across the province of Northern Ireland were sampled (located in Enniskillen Craigavon, Lisburn and Belfast), see S.I. S1 File “all sample details” tab for a breakdown. The bulk of the data were labelled as being from the EU or produced in UK or Belgium as their source of origin, only 4 products actually stated the country of origin of the rice (one Italian, 2 Japanese, 1 Thai), reported in “product averages” tab of S.I. S1 File. Details of the UK 2016 and 2014 survey are given in Signes-Pastor et al. [7].

### Sample preparation and digestion

Samples were dried in a Christ LD freeze dryer, powderized on a rotary ball mill (Retch PM 100 planetary ball mill) using a zirconium oxide lined milling chamber and 20mmØ zirconium oxide marbles. From each powdered sample, ~100mg was accurately weighed out using OHAUS- Discovery digital weighing scales and transferred into labelled 50ml polypropylene centrifuge tubes and the precise weight recorded. Three additional tubes were used as blanks and 3 for CRMs. For As speciation, NIST SRM 1568b rice flour was used as the CRM as this contains certified levels of several relevant As species. Then 1% nitric acid (10mls) was pipetted into each tube and swirled briefly. This was allowed to soak overnight. Centrifuge tubes were then placed into the carrousel for the CEM Mars 6 1800W microwave digester heated up to 95°C gradually by heating from ambient to 55°C in 5 minutes, hold for 10 minutes, heat to 75°C, hold for 10 minutes, heat to 95°C in 5 minutes, hold for 30. After cooling each tube was made up to the final weight (10g) with 1% nitric acid. The precise weight of this was recorded. Tubes were centrifuged using a Sorvall Legend RT centrifuge which was set to 3500 rpm for 15 minutes at 20°C.

### Ion chromatography fractionation for speciation

From each centrifuge tubes, 700μl was transferred to a 1ml pp vial and, finally, 7μl of Prolabo Analar Normapur hydrogen peroxide 30% (or similar) was added using a pipette, and the vials were thoroughly mixed. Vials were arranged into trays according to a predetermined random run order and placed into the auto-sampler. A Thermo Dionex IC5000 Ion Chromatograph (IC) system was used including Dionex IonPac AS7 RFIC analytical column (2x250mm) and Dionex AG7 guard column. The mobile phase A consisted of 20mM ammonium carbonate in deionised water and the mobile phase B consisted of 200mM ammonium carbonate in deionised water. The flow rate was 0.3 ml/min using the following gradient program: 100% mobile phase A when time = 0 mins, followed by a linear change to 100% mobile phase B when time = 10 mins and finally followed by a linear change to 100% mobile phase A when time = 10.5 mins followed by 2minutes equilibration, total analysis time 12.5 minutes.

### Arsenic analysis by ICP-MS

The IC was connected to an ICP-MS (Thermo ICAP-Q) detector. The ICP-MS operating conditions were: Forward RF power- 1550W; Nebuliser gas flow- ~1L/min, nebuliser sample flow rate- ~0.35ml/min. Helium was used as a collision gas at a flow rate of 4.5 ml/min., and the sole element measured was As at a mass of 75. A mixed species solution was made up containing: AB- arsenobetaine (EC IRMM BCR626); DMA- dimethylarsinic acid (Supleco [Merck]); MMA- monomethlyarsonic acid (Chem Service); Tetra- tetramethylarsonium acid (a gift from Joeg Feldmaa, University of Aberdeen); AsIII arsenite (Sigma-Aldric [Merck]); AsV- sodium, dibasic, arsenate heptahydrate (Sigma-Aldrich) at 1 μg/L as retention time check. Five standards were made up including one blank, all in 1% HNO_3_, in the range 0-5ppb using DMA as the calibrant species, and all species concentrations were measured against this calibration.

### Procedure for statistical analysis

For statistical analysis medians for each product class, porridge, crackers or cereals, are were calculated, with either a Mann-Whitney unpaired t-test or Kruskal-Wallis non-parametric one-way ANOVA used, to compare medians, using the statistical package GraphPad Prism v.6. Linear regression analysis was used to compare inorganic arsenic content to percentage rice in each product.

## Results

Quantification of NIST-1568b CRM, N = 4, was 103.4% (RSD 8.3%), 94.5% (RSD 10.4%), 83.7% (RSD 12.7) and 96.6% (RSR 9.3%) for DMAA, MMAA, inorganic arsenic and sum of species, respectively. The limits of detection (LoD) for inorganic arsenic and DMAA was 1 μg/kg. Where samples were below LoD, half LoD was assigned for statistical consideration. The average inorganic arsenic and DMAA concentration was calculated for each sample product, reported for UK samples in S1 File.

**Fig 1:**
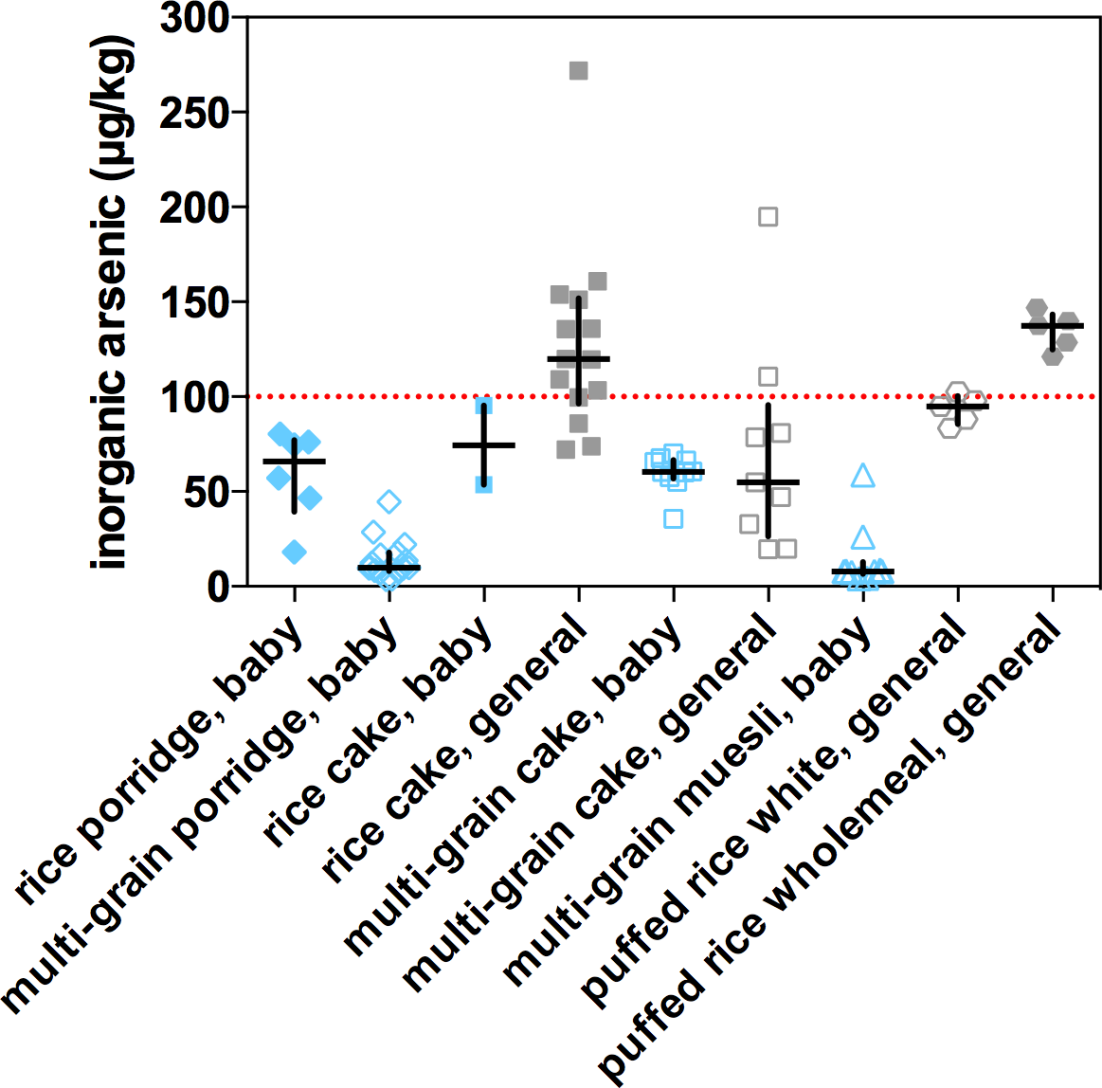
Median inorganic arsenic concentrations. Median concentrations of the average inorganic arsenic content of individual rice products (reported in S1 File, “product medians” tab) specifically for infants (shaded blue) and for the general market (shaded grey). Diamonds are for porridge (pure baby rice, n = 6; multi-grain porridge, n = 15), squares for cakes (baby rice cake, n = 2; general rice cake, n = 14, baby multi-grain cake, n = 10; general multi-grain cake, n = 9), triangles for muesli (multi-grain muesli, 10), hexagonals for puffed rice (white, n = 5, wholemeal, n = 5). The median values for each product class are reported in S1 File, “product class medians” tab.

**Fig 2:**
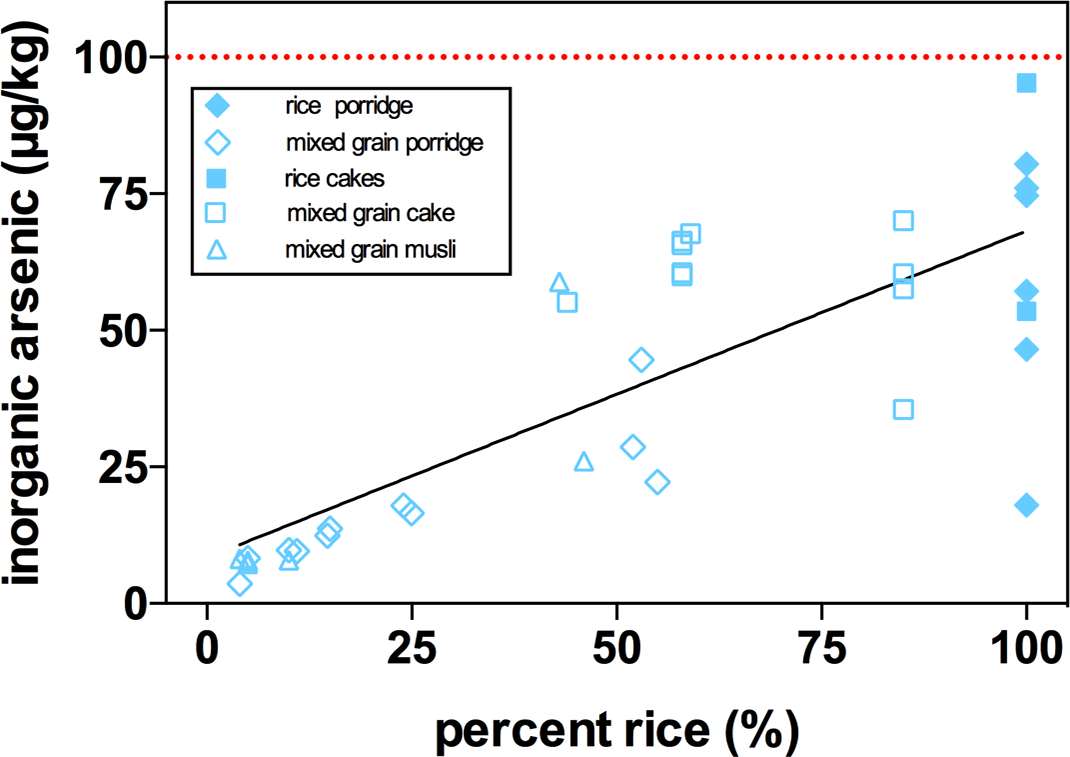
Correlation between rice content of infant foods and inorganic arsenic content. Correlation between rice content of infant foods and inorganic arsenic content averages for individual products with medians reported in SI S1 File, “product averages” tab. Diamonds are for porridge (pure rice, n = 6; mixed grain porridge, n = 15), squares for cakes (rice cake, n = 2; mixed grain cake, n = 10), triangles for mixed grain muesli (n = 10).

The median values for arsenic species concentrations, and N, for the 2017 survey are reported in the “product class medians” tab of S1 File. A range of UK children’s food products, and their generic, i.e. not specifically labelled for the consumption of young children, equivalents, inorganic arsenic contents are presented in Fig 1. There were a range of products for babies that contain rice and these can be considered pure rice products, cakes and porridge, and multi-grain products, cakes, porridges and muesli. For generic products, cakes, both pure rice, and puffed rice, white and wholemeal, were widely available. Multi-grain products usually included maize, but could include quinoa, oats and potato in various quantities (S1 File, “all samples detail tab”). All products specifically for children fell under the 100 μg/kg EU inorganic arsenic standard, with medians also below this threshold. For baby porridges, pure rice had a median of 66 μg/kg, as compared to 10 μg/kg for multi-grain. A similar difference was observed between children’s pure rice cakes and multi-grain, 60 versus 8 μg/kg inorganic arsenic, respectively. There were only 2 brands of pure baby porridge available in the stores surveyed. The differences between rice versus multi-grain were both highly significant (P<0.0001) for both porridges and cakes. Multi-grain cakes for children were not significantly lower than for adults, but the variance was lower. Generic rice cakes had a median above the EU inorganic arsenic standard at 120 μg/kg. These crackers tended, 13 out of 14, to contain wholemeal rice. Generic white (milled before puffing) puffed rice had a median inorganic arsenic content of 95 μg/kg, while wholemeal puffed rice was the highest of any product class at 140 μg/kg inorganic arsenic. When inorganic arsenic content of baby foods was correlated with rice content of the product, where that content was stated on the packaging (S1 File, “all sample details” tab), there was a highly significant correlation (P<0.0001) with an R^2^ of 0.632 (Fig 2). The multi-grain porridges and mueslis, in particular, could have a very low rice content, and a correspondingly low inorganic arsenic content.

**Fig 3:**
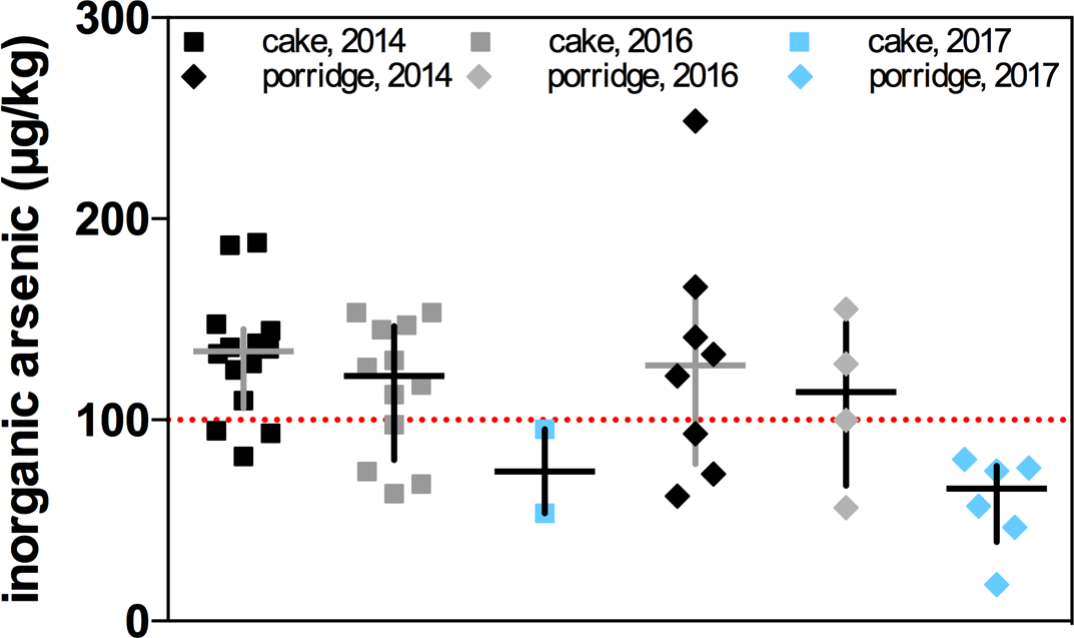
Median concentrations of the average inorganic arsenic content in individual infant rice products from 2014–2017. Median concentrations of the average inorganic arsenic content in individual infant rice products sampled from 2014 (cake, n = 14; porridge, n = 8), 2016 (cake, n = 12; porridge, n = 4) and 2017 surveys (cake, n = 2; porridge, n = 6).

When year of sampling is considered for cakes and porridge, infant mueslis being a relatively new innovation, at least on a mass distribution basis for UK baby foods, inorganic arsenic concentrations have dropped in 2017 (Fig 3). The amount of baby rice cakes found on the shops surveyed was only 2 brands in 2017 compared to previous years when the there was a larger variety of brands and formulations found, 14 in 2014, 12 in 2016. While the median in 2017 was below 100 μg/kg, the fact that there were only 2 brands precludes statistical analysis. Rice porridge was more prolific as a product and had a median of 66 μg/kg in 2017, compared to 114 μg/kg in 2016 and 127 μg/kg in 2014. These medians for rice porridge were statistically significant, P = 0.0393.

## Discussion

The findings presented here for the UK market clearly indicate that there has been a considerable shift in baby food formulation with respect to ensuring those products fall below the 100 μg/kg EU inorganic arsenic standard [12]. The approaches taken by manufactures are three-fold. For the 1^st^ approach, pure rice products, such as porridges and crackers, it appears that low inorganic rice has been specifically sourced for formulation. The 2^nd^ approach appears to be a lower representation of pure rice products being produced/available, though this would take manufacturer survey to quantify. The 3^rd^, and most radical, approach is to blend rice with other gluten free grains, as well as the introduction of multi-grain mueslis for infants, to dilute out the inorganic arsenic in rice. Although the manufacturers have not stated or advertised this approach, it seems that they have, moved to meet EU standards by using dilution of the rice content of infant foods. However, diversification of grains in the diets of children needs to take account of all risks, not just inorganic arsenic. For baby foods, the rice alternatives favoured by manufacturers are maize, oats, quinoa and potato, all gluten free, as preferred, particularly at weaning, in the infant market. Maize and oats can be problematic with respect to their mycotoxin content, and surveys of infant cereals show that relatively high percentages had mycotoxin concentrations exceeding those allowed by EU standards [17–20]. Rice is, generally, low in mycotoxins [21].

This UK reduction in rice based infants’ foods will lead to lower levels of inorganic arsenic exposure in children, however, more can be done. It is important to note that the EU standard of 100 μg/kg inorganic arsenic [12] was not set following a risk assessment, in fact the logic of 100 μg/kg inorganic arsenic as a standard has not been explained, and the same as for the 200 μg/kg inorganic arsenic standard for non-infant rice products [12]. The rational of setting the infant standard at half that for adults seems arbitrary as young children are effectively exposed to 3-times more arsenic than adults due to their high food consumption rate on a body mass basis [7, 13], and the fact that children appear more sensitive to inorganic arsenic than adults [3–6]. Even a primitive risk assessment, based on food consumption rates, would suggest that the children’s thresholds for maximum concentrations of inorganic arsenic in rice should be at least a 1/3 of that for adults, though even lower would be better. A illustration of how the infant foods regulation is not well thought though can be achieved by considering inorganic arsenic intakes by babies from rice based products. Assuming a 20g portion for a 9.25 kg (1-year old) child [22], a 100 μg/kg EU inorganic arsenic rice based infants food limit, this means a child will receive a dose of 0.22 μg per kg body weight per sitting. Assuming consumption of 1L a typical daily consumption) of 3 μg/L water for a 70 kg adult person, dose of 0.042 μg/kg bodyweight per day (μg/kg/d), inorganic is the estimated 4–7 in 10,000 excess bladder and lung cancer life-time exposure risk, considered baseline [23]. The EU, US and WHO [24] water standards are 10 μg/L, and this same body weight arsenic consumption for a 70 kg adult at 10 μg/L consuming 1L per day is 0.142 μg/kg/d. A single portion of a rice based meal at the EU limit will take the child over the adult 3 μg/L water ingestion by 5-fold, and at the 10 μg/L water standard by 1.5-fold, given that babies may be fed more than one rice based meal per day. While 10 μg/L is the EU, US and WHO standard, few around the world are actually exposed to this concentration of inorganic arsenic in their drinking water, yet children are exposed daily substantially more inorganic arsenic, on a body weight dose basis, from rice based foods. While the epidemiological modelling is or life-time exposures [23], children are known to be more susceptible to inorganic arsenic than adults [3–6,11]. The rational for setting stricter EU, and elsewhere, standards for inorganic arsenic in rice based baby foods, and adult foods as rice is again here a dominant exposure route, is compelling, as we have previously highlighted [24]. Our results show that attaining lower inorganic arsenic standards in infant foods is readily achievable, i.e. the manufacturers have already done this, with the median concentration in multi-grain cereals porridge that contain rice as an ingredient being 9.75 μg/kg, a tenth lower than the EC [12] standard. Thus, the move away from away from pure rice cereal products, and the sourcing of low arsenic rice appears to be a positive step for the UK market.

The WHO, although it does not have an infant standard, has set a standard for polished rice, at the same value as the EU, 200 μg/kg inorganic arsenic [25]. Again, this WHO standard was not set on a risk assessment basis, but at least the logic of the WHO decision was outlined in their discussion documents [25]. The WHO discussions around where to set an inorganic arsenic in rice standard explicitly state that the standard was set so as to keep most of the rice in the global food-chain in circulation [25]. That is, the WHO standard was set on economic grounds, supposedly using the As Low as Reasonably Practical (ALARP) principle [25]. Our UK findings presented here confound this WHO ALARP assessment interpretation as pure baby rice can be sourced that is below 100 μg/kg, half the WHO [25] standard, for a commercial product. It is clear that tighter law setting, 100 μg/kg for the EU for infant foods vs. 200 μg/kg generically for EU and WHO, is an incentive for lowering inorganic arsenic in the diet. Given that inorganic arsenic is a non-threshold carcinogen [22, 26], i.e. that every dose carries a carcinogenic risk, exposures to babies, and adults, should be minimised.

Other strategies, besides dilution with other grains, could be employed to further lower inorganic arsenic content of rice based baby foods. Inorganic arsenic is readily cooked out of rice, at removal rates of 80% [26]. As infant products are normally precooked, inorganic arsenic removal at the food processing stage is a tractable option. Also, rice for infant foods, could be sourced from low arsenic rice regions [8]. This appears to what has occurred for pure rice infant porridges and crackers in the UK from the results presented here.

While the implementation of a 100 μg/kg inorganic arsenic standard for baby foods has had a positive impact of reducing the exposure of infants to arsenic, the law does not take into account parents who will feed their children foods classified as not being labelled specifically for young children [12]. Such foods include rice grain, and parents who make their babies meals from fresh ingredients will expose their children to higher inorganic arsenic levels than if pre-packaged foods labelled as being for children were given as a meal instead. Also, as illustrated by the findings here. If non-infant food labelled rice breakfast cereals or rice crackers are given to young children, then their exposures will be higher than giving them the equivalent child-food product. Puffed rice is off particular concern as it is a food widely given to children as a breakfast cereal, but the EU law allows it to have the highest inorganic arsenic concentration of any rice product, 300 μg/kg [12].

As rice is not just a problem for infants, it being a chronic carcinogen with risks measured over life-times [22, 26], if inorganic arsenic exposures from rice can be reduced for infants they also can be reduced for adults. Again, sourcing is a solution for non-rice growing countries, such as the northern European countries focussed on in this study, or from simple changes in cooking, such as pre-soaking and using a large water: rice (ratio) [27]. For countries where rice is the subsistence carbohydrate source, as well as changing cooking, rice processing, such as parboiling practice [28], can also make an impact. Diversification of diet is also an option, and this is would generally help the nutritional health of the populace in countries with rice subsistence diets [29]. Agronomic solutions to producing low arsenic rice, for various reasons, have not emerged [30], but may do so in the future.

## Supporting information

S1 File Sample information and arsenic speciation for individual samples, and means per sample type. The file contains an Excel sheet with data tabulated under the tabs: “product class medians”, “product averages” and “all sample details”.
(XLSX)
